# Pd single atoms on g-C_3_N_4_ photocatalysts: minimum loading for maximum activity†

**DOI:** 10.1039/d4sc08589b

**Published:** 2025-02-12

**Authors:** Velu Jeyalakshmi, Siming Wu, Shanshan Qin, Xin Zhou, Bidyut Bikash Sarma, Dimitry E. Doronkin, Jan Kolařík, Miroslav Šoóš, Patrik Schmuki

**Affiliations:** a Department of Materials Science WW4-LKO, Friedrich-Alexander-University of Erlangen-Nuremberg Martensstrasse 7 91058 Erlangen Germany schmuki@ww.uni-erlangen.de; b Department of Chemical Engineering, University of Chemistry and Technology Technická 3 Prague 160 00 Czech Republic; c Laboratoire de Chimie de Coordination (LCC), CNRS, Université de Toulouse, INPT, UPR 8241 205 Route de Narbonne 31077 Toulouse Cedex 4 France; d Institute of Catalysis Research and Technology, KIT Hermann-von Helmholtz Platz 1 76344 Eggenstein-Leopoldshafen Germany; e Regional Centre of Advanced Technologies and Materials Šlechtitelů 27 78371 Olomouc Czech Republic

## Abstract

Noble metal single atoms (SAs) on semiconductors are increasingly explored as co-catalysts to enhance the efficiency of photocatalytic hydrogen production. In this study, we introduce a “spontaneous deposition” approach to anchor Pd SAs onto graphitic carbon nitride (g-C_3_N_4_) using a highly dilute tetraaminepalladium(ii) chloride precursor. Maximized photocatalytic activity and significantly reduced charge transfer resistance can be achieved with a remarkably low Pd loading of 0.05 wt% using this approach. The resulting Pd SA-modified g-C_3_N_4_ demonstrates a remarkable hydrogen production efficiency of 0.24 mmol h^−1^ mg^−1^ Pd, which is >50 times larger than that of Pd nanoparticles deposited on g-C_3_N_4_*via* conventional photodeposition. This significant enhancement in catalytic performance is attributed to improved electron transfer facilitated by the optimal coordination of Pd SAs within the g-C_3_N_4_ structure.

## Abstract 2

g-C_3_N_4_ modified with Pd single atoms – decorated by reactive deposition – exhibits remarkable photocatalytic hydrogen production efficiency with a low loading of 0.05 wt%, far outperforming g-C_3_N_4_ decorated with Pd nanoparticles.

## Graphical abstract

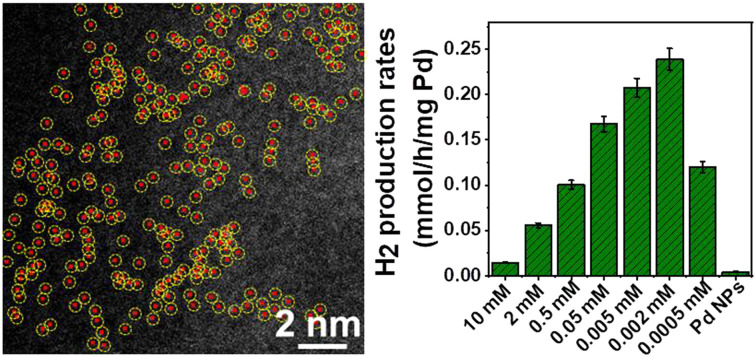

## Introduction

In recent years, the demand for hydrogen as a clean renewable energy carrier has grown rapidly, driven by the need to replace traditional fossil fuels. Photocatalytic water splitting is a promising and most elegant way to directly convert solar energy into clean, renewable hydrogen (H_2_).^1,2^ Since the pioneering work of Fujishima *et al.*, titanium dioxide (TiO_2_) has become the most extensively studied material for this purpose.^3–5^ More recently, graphitic carbon nitride (C_3_N_4_) is being increasingly explored as it shares many advantageous features with TiO_2_, such as high stability, abundance, and a suitable band structure for water splitting but provides the key advantage of strong visible light absorption, resulting in significantly enhanced solar light utilization.^6–9^

Nevertheless, due to strong kinetic hindrance for the photocatalytic hydrogen evolution reaction (HER) on the C_3_N_4_ surface, an enhanced efficiency highly depends on the development of highly active co-catalysts – typically noble metals such as Pt, Pd, and Rh need to be deposited on the semiconductor surface to obtain a reasonable H_2_ production rate. Despite efforts made using non-precious metal HER electrocatalysts,^10^ noble metal-based materials remain nearly irreplaceable due to their superior activity and chemical stability. However, the scarcity and high cost of these metals pose significant challenges to the large-scale application of these noble metal-based co-catalysts. This has prompted considerable efforts to minimize noble metal usage while retaining high catalytic performance. In this context, single-atom catalysts have garnered wide attention due to their maximized atom utilization efficiency.^11–13^

As for many other semiconductors and also for C_3_N_4_ substrates, a wide range of noble metal SAs have been successfully deposited as nanoparticles, clusters or SA, and accordingly improvement in photocatalytic H_2_ production has been reported.^14–21^ In search of the most active SA species, Akinaga *et al.* conducted a remarkable study on ten transition metal elements, including Cu, Ni, Pd, Pt, Rh, Ru, Ag and Au, as SAs anchored on g-C_3_N_4_. Among the metals tested, Pd demonstrated significantly higher hydrogen evolution activity compared to other precious metals such as Pt, Rh as well as other transition metals.^14^ The authors ascribed the superior activity of Pd on C_3_N_4_ to the suitable electronic structure of this metal on C_3_N_4_. In their work, Akinaga *et al.* used a photodeposition approach to achieve a relatively high SA loading (>0.5 wt%).

However, for many semiconductors, the activity of SAs in photocatalysis is extremely dependent on the deposition approach.^13,21–23^ Namely, for Pt SAs on TiO_2_, it was reported that “reactive” deposition leads to highly active SA configurations that can provide maximized H_2_ production, *i.e.*, a very high catalytic efficiency can be reached at very low noble-metal loading. This approach relies on the surface reaction of highly dilute solutions of suitable noble-metal precursors.^24–27^

In the present work, we first explore various Pd precursors for the feasibility of a reactive SA attachment on C_3_N_4_. We find that tetraaminepalladium(ii) chloride – Pd(NH_3_)_4_Cl_2_ as a Pd precursor solution with C_3_N_4_ allows for an adjustable Pd SA loading with a wide range of deposition concentrations from 0.04 wt% to 0.75 wt%. Our results show that by the reaction of a minimal amount of a 0.05 mM precursor, maximum photocatalytic efficiency can be obtained. The photocatalytic hydrogen production activity of such Pd SA-decorated C_3_N_4_ achieves a normalized H_2_ production activity of 0.24 mmol h^−1^ mg^−1^ Pd, which is 55 times higher than that observed with Pd nanoparticle-decorated C_3_N_4_ at an effective loading that is more than 10 times lower than that typically reported in the literature for Pd on C_3_N_4_. The superior activity of Pd SAs/C_3_N_4_ is attributed to the strong coordination of Pd SAs within the C_3_N_4_ structure, forming a highly stable and catalytically effective configuration that drastically reduces the charge transfer resistance for the HER. These results illustrate how a refined anchoring of SAs on substrates can enable more cost- and production-effective use of precious metals in photocatalysis.

## Results and discussion

**Fig. 1:**
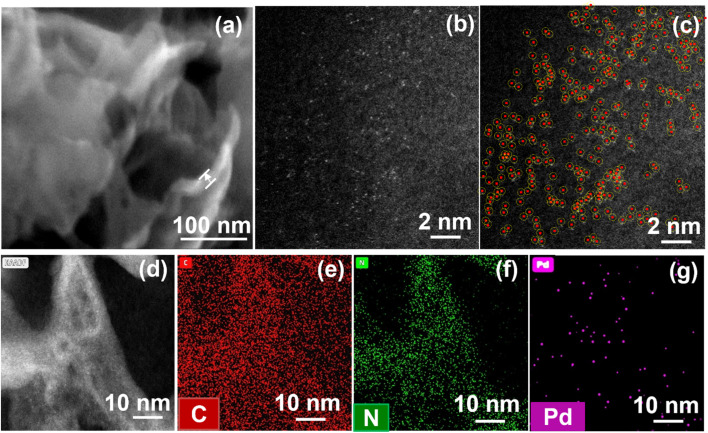
(a) SEM image, (b) original HAADF-STEM image, (c) the HAADF-STEM image with individual Pd SAs highlighted by red dots and yellow circles, (d) HAADF-STEM image and the corresponding EDS mapping (e) C, (f) N, and (g) Pd of Pd SAs/C_3_N_4._

Nanosheets of g-C_3_N_4_ were synthesized using a thermal polycondensation method starting from an equimolar mixture of melamine and dicyandiamide, followed by thermal exfoliation, as described in the literature.^28–30^ In order to explore the feasibility of direct deposition of a (reactive) SA such as Pd on C_3_N_4_, we examined different precursor species, namely tetraamminepalladium(ii) chloride (Pd(NH_3_)_4_Cl_2_), palladium(ii) chloride (PdCl_2_) and ammonium hexachloropalladate(iv) ((NH_4_)_2_[PdCl_6_]). To investigate the reactive deposition behavior, we used three different Pd precursors at a concentration of 2 mM to decorate Pd on C_3_N_4_. We then evaluated the general deposition behavior with electron microscopy and XPS and also evaluated the photocatalytic H_2_ production performance. Among the samples, XPS results reveal that both PdCl_2_ and (NH_4_)_2_[PdCl_6_] lead to relatively high Pd loadings (>1 at%) (Fig. S1a and b†); however, the strong Cl 2p signals in the XPS spectra (Fig. S1c†) indicate that most of the Pd precursor did not react with C_3_N_4_, *i.e.*, the precursor is just physically adsorbed on the C_3_N_4_ surface. In the SEM images of these two samples (Fig. S2†), obvious Pd nanoparticles can be seen, due to the agglomeration caused by high loading. In contrast, the Pd(NH_3_)_4_Cl_2_ sample shows no visible metal nanoparticle formation in SEM (Fig. 1a), non-metallic Pd position in XPS (Fig. S1a†) and no detectable Cl 2p signal (Fig. S1b†), indicating a complete reaction of this particular precursor with the C_3_N_4_ surface.

Fig. 1a shows the SEM image of Pd-deposited g-C_3_N_4_ (Pd SAs/C_3_N_4_) using Pd(NH_3_)_4_Cl_2_ at a concentration of 0.002 mM, and Fig. S3† shows the SEM image of neat g-C_3_N_4_. The introduction of Pd SAs does not affect the morphology of C_3_N_4_ – both samples show a sheet-like structure with a thin layer thickness of approximately 16 nm. The high-angle annular dark-field scanning transmission electron microscopy (HAADF-STEM) image of Pd SAs/g-C_3_N_4_ is shown in Fig. 1b and c, which confirms the presence of individual Pd atoms (highlighted in Fig. 1c with red dots and yellow circles). Also, in the HAADF-STEM image (Fig. 1d), there are no observable Pd agglomeration on the g-C_3_N_4_ surface. Energy-dispersive X-ray spectroscopy (EDX) mapping (Fig. 1e–g) further proves the uniform dispersion of Pd SAs throughout the g-C_3_N_4_ structure. The density of Pd SAs was calculated as 1.6 × 10^6^ μm^−2^ from HAADF-STEM images shown in Fig. S4.†

**Fig. 2:**
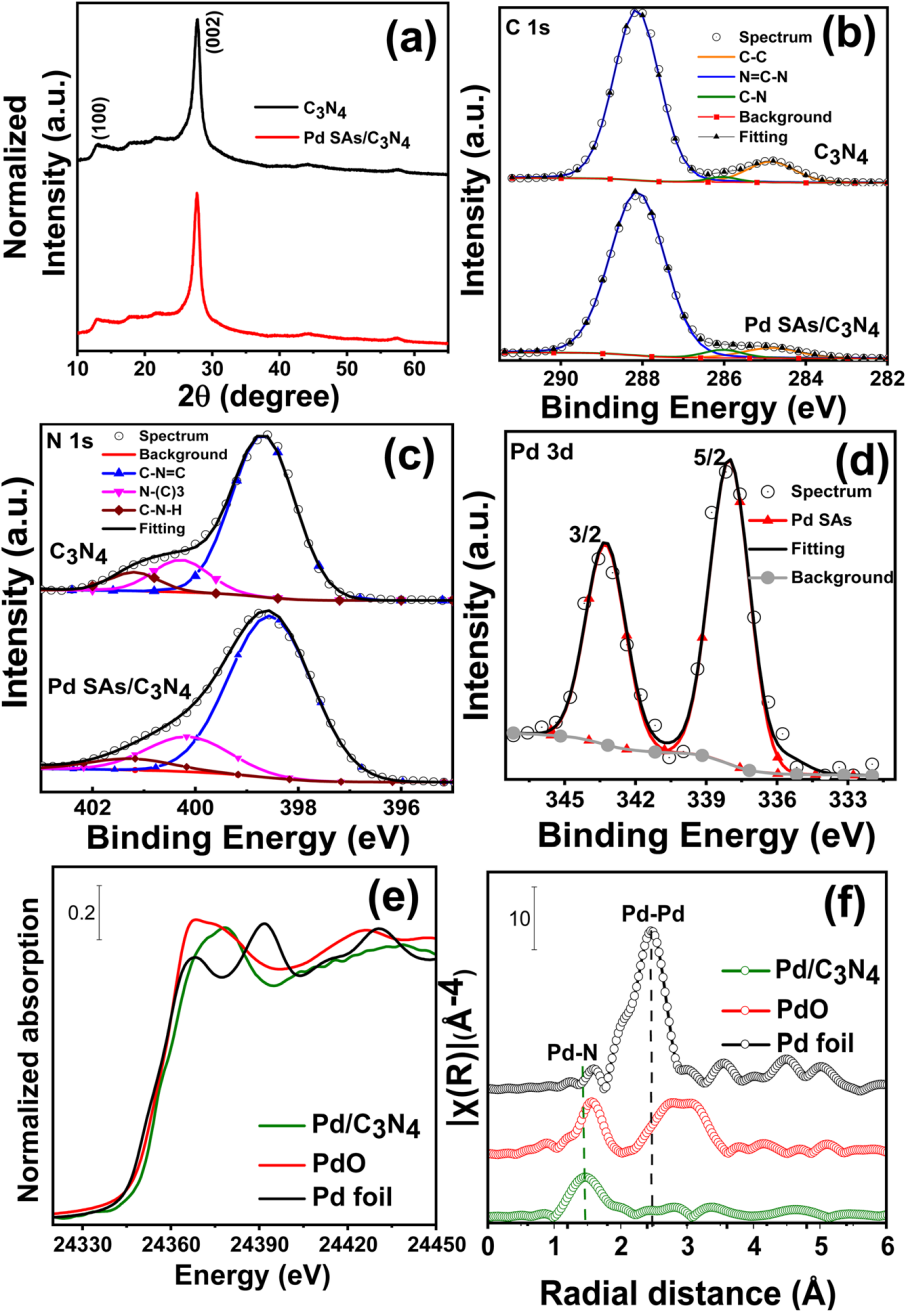
(a) X-ray diffraction pattern, (b–d) XPS spectra of (b) C 1s, (c) N 1s and (d) Pd 3d for C_3_N_4_ and Pd SAs/C_3_N_4_, (e) XANES spectra of Pd/C_3_N_4_ at Pd K-edge, and (f) Fourier transform extended X-ray absorption fine structure (FT-EXAFS) spectra of Pd SAs/C_3_N_4_.

X-ray diffraction (XRD) patterns of g-C_3_N_4_ and Pd SAs/g-C_3_N_4_ are presented in Fig. 2a. Both samples display two distinct diffraction peaks at 13° and 27.6°, corresponding to the (100) and (002) crystal planes of g-C_3_N_4_, respectively.^31^ Notably, no diffraction peaks related to metallic Pd are observed in the Pd SAs/g-C_3_N_4_ sample (as is expected for the SA-decorated sample).^14^

X-ray photoelectron spectroscopy (XPS) was utilized to investigate the chemical state of g-C_3_N_4_ and Pd SAs on C_3_N_4_ (Fig. 2b–d and Table S1†). The high-resolution C 1s XPS spectrum (Fig. 2b) of both samples can be fitted by three peaks at 284.7 eV, 286.2 eV, and 288.1 eV corresponding to C–C, C–N and N–C=N of the heptazine ring carbon structure, respectively.^16^ The fitted N 1s spectra (Fig. 2c) exhibit peaks at 398.7 eV (N1), 400 eV (N2), and 401.4 eV (N3), representing the sp^2^ hybridized aromatic two-coordinated (N2c) nitrogen of the triazine unit (C=N–C, *i.e.*, pyridinic N) and three coordinated (N3c) bridging N atoms connected to carbon as N–(C)_3_ groups and amino functional (C–NH/NH_*x*_) groups respectively.^16,32,33^ The Pd 3d spectrum of Pd SAs/C_3_N_4_ shows two peaks (Fig. 2d), doublets at 337.6 and 342.9 eV corresponding to Pd^*δ*+^ 3d_5/2_ and Pd^*δ*+^ 3d_3/2_ (0 < *δ* < 2), respectively.^16^ For comparison, Pd nanoparticles were deposited on g-C_3_N_4_ (Pd NPs/C_3_N_4_) using an established photodeposition method described in the literature.^34,35^ SEM images of Pd NPs/C_3_N_4_ (Fig. S5†) clearly show distinct Pd nanoparticles on the C_3_N_4_ surface with their typical diameter in the range of 7–15 nm. For this sample, the Pd 3d XPS spectra (Fig. S6†) of Pd NPs/C_3_N_4_ exhibit doublets at 335 eV and 340 eV, which are typically attributed to metallic Pd^0^.^33^

The nature of Pd species was further investigated by X-ray absorption spectroscopy (XAS) and diffuse reflectance infrared Fourier transform spectroscopy (DRIFTS) measurements. Fig. 2e and f show the absorption near-edge structure (XANES) and extended X-ray absorption fine structure (EXAFS) spectra of Pd SAs/C_3_N_4_. The X-ray absorption spectrum of Pd SAs/C_3_N_4_ measured at the Pd K-edge (24 350 eV) indicates the presence of Pd atoms that are non-metallic and carry a positive charge.^15,16,20^ The EXAFS analysis and the corresponding Fourier transformed (FT) radial distribution function of Pd SAs/C_3_N_4_ show a peak at approximately 1.5 Å (without phase correction), attributed to the Pd–N bond, and no obvious scattering is observed for the metallic Pd–Pd bonding.^16,20^ CO-DRIFT spectra of Pd SAs/C_3_N_4_ (Fig. S7†) show a CO vibrational peak at 2125 cm^−1^, which is characteristic of linearly bonded CO on a Pd single site (usually Pd^2+^).^36,37^ These results are well in line with the XPS results, *i.e.*, Pd SAs are N-coordinated in C_3_N_4_ with an oxidation state ≈ 2.

**Fig. 3:**
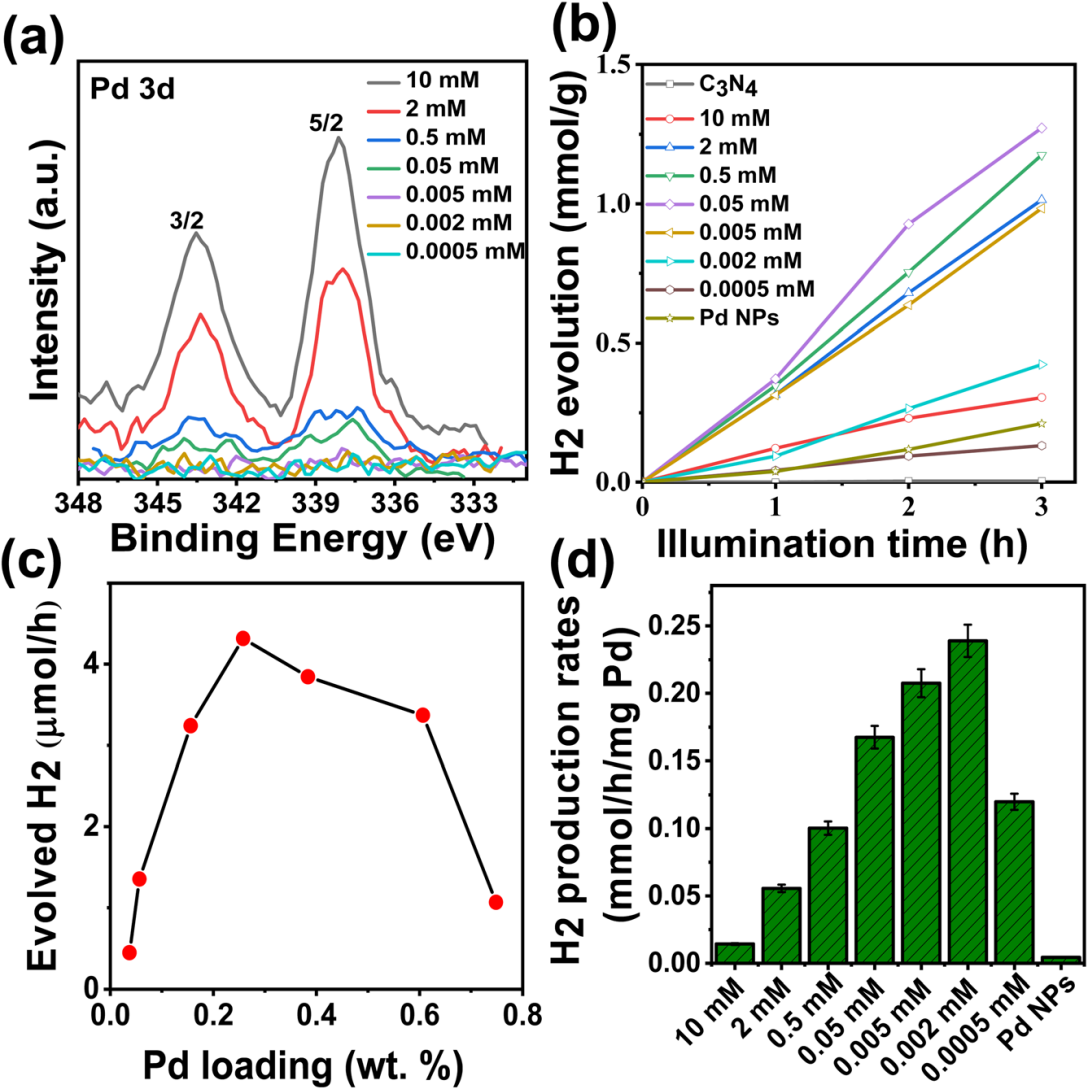
(a) Pd 3d XPS spectra of Pd SAs/C_3_N_4_ at different concentrations of tetraaminepalladium chloride, (b) photocatalytic H_2_ evolution, (c) evolved H_2_ at different Pd SA loadings, and (d) normalized H_2_ evolution rates for different concentrations Pd SAs using tetraaminepalladium chloride.

We then used the above deposition approach to place Pd SAs from Pd(NH_3_)_4_Cl_2_ solutions in the concentration range of 0.0005 mM to 10 mM on C_3_N_4_. Fig. 3a shows the Pd 3d XPS spectra for these Pd SAs loaded on C_3_N_4_ samples. Notably, neither metallic Pd peaks nor Cl 2p peaks (Fig. S8†) are observed under any of the deposition conditions. Instead, the incorporation of Pd SAs is evident across all samples, as indicated by the Pd 3d doublet peaks at 337.6 and 342.9 eV. The XPS data align with the SEM images shown in Fig. S9,† where no Pd nanoparticles are observed in any of the samples, even at the highest precursor concentration of 10 mM.

The bulk loading of the samples prepared using different concentrations of Pd(NH_3_)_4_Cl_2_ solutions was further quantified by atomic absorption spectroscopy (AAS) – the results are shown in Table S1.† As the concentration of the Pd precursor increases, the loading of Pd SAs increases (Fig. S10†), which is consistent with the XPS data (Table S2†). For the highest precursor concentration of 10 mM, the Pd SA loading reaches 0.75 wt%.

We then examined for all samples the photocatalytic H_2_ evolution using a 365 nm LED light source with an intensity of 65 mW cm^−2^ and an aqueous solution of 10% triethanolamine (TEOA) as a hole scavenger.^19,38,39^ From the results shown in Fig. 3b, it is evident that the decoration of either Pd NPs or Pd SAs significantly enhances the H_2_ production activity compared to bare C_3_N_4_. In the concentration range of 0.0005 mM to 0.05 mM, corresponding to Pd SA loadings from 0.04 wt% to 0.26 wt% (Fig. S10†), the photocatalytic H_2_ production activity increases with loading (Fig. 3c). It is worth mentioning that Pd SAs deposited through our method, utilizing the Pd(NH_3_)Cl_2_ precursor, lead to the notable finding that even a minimal loading of 0.05 wt% outperformes Pd NPs synthesized *via* photodeposition, the latter having a much higher loading of 1.5 wt% (Table S2†). As a side note, this loading is also much more efficient than using PdCl_2_ and (NH_4_)_2_PdCl_6_ precursors (see Fig. S11†). Also, in this comparison, the photocatalytic H_2_ production results show that an SA deposition approach using Pd(NH_3_)_4_Cl_2_ leads to much higher activity, despite the significantly lower Pd loading compared with PdCl_2_ and (NH_4_)_2_PdCl_6_.

In general, the data clearly show that the H_2_ production amount increases with Pd precursor concentration loading until reaching a maximum at 0.05 mM (with a Pd SA loading of 0.26 wt%). Beyond this point, a further increase in Pd loading does not increase the activity any further but even leads to a slight drop in the activity and in the 10 mM case a very obvious decrease (Fig. 3c). This is likely due to the decrease in the density of Pd SAs and the formation of Pd agglomerates, which may increase charge recombination.^40^

To further assess and compare the effectiveness of Pd as a co-catalyst in both single-atom and nanoparticle forms, we normalized the data from Fig. 3b relative to Pd loading (Table S1†); the results are shown in Fig. 3d. The analysis reveals that the highest mass-specific photocatalytic efficiency, resulting in an H_2_ production rate of 0.24 mmol h^−1^ mg^−1^ Pd, is achieved with 0.002 mM Pd precursor (0.05 wt%). This efficiency is 55 times higher than that obtained through conventional photodeposition of Pd nanoparticles on g-C_3_N_4_, highlighting the superiority of our reactive deposition method for optimizing photocatalytic H_2_ production. The exceptional performance of low Pd SA loading on g-C_3_N_4_, prepared using our direct deposition method, is evident when compared to other Pd SA-loaded g-C_3_N_4_ structures reported in the literature for photocatalytic H_2_ generation. As shown in Table S3,† our work demonstrates the highest photocatalytic hydrogen evolution per Pd atom. Notably, even when compared to studies with similar or higher Pd SA loadings, the Pd SAs obtained through our reactive deposition method using Pd(NH_3_)_4_Cl_2_ exhibit the highest efficiency.

**Fig. 4:**
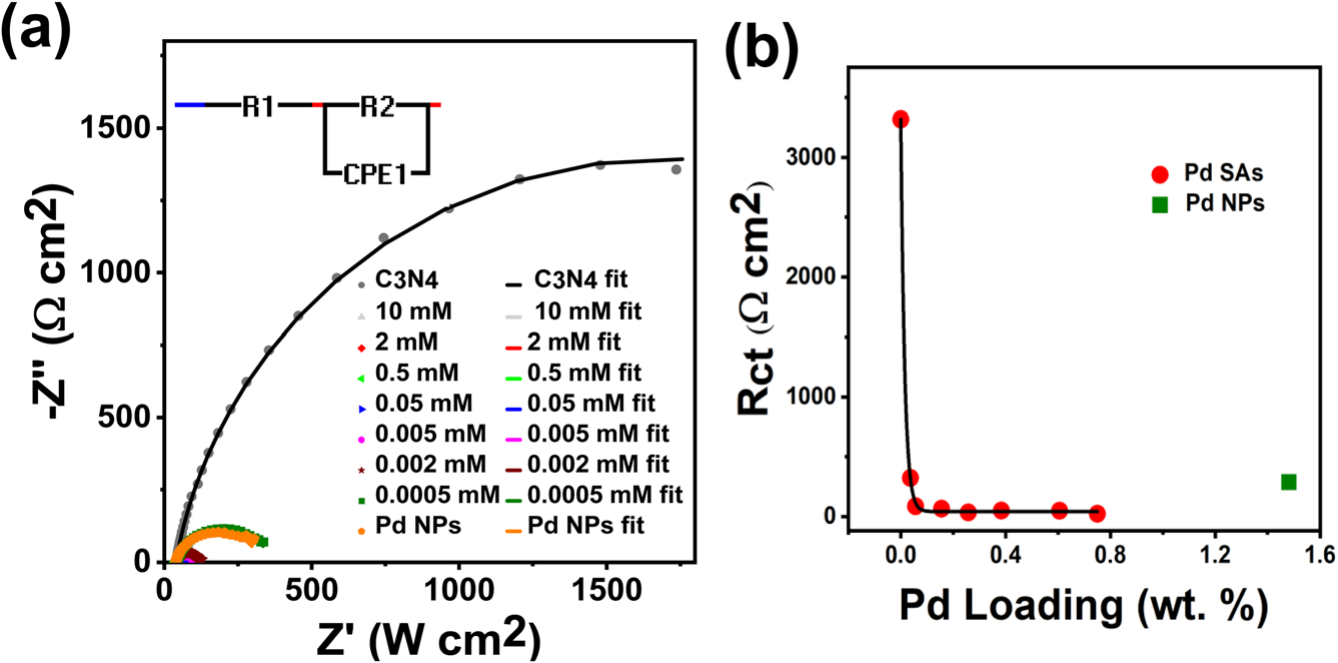
(a) EIS plots of C_3_N_4_, Pd SAs/C_3_N_4_ and Pd NPs/C_3_N_4_ at the voltage of −0.5 V (*vs.*.Ag/AgCl) in 0.1 M Na_2_SO_4_ aqueous electrolyte. The equivalent circuit model used for fitting is depicted in the inset of (a). (b) *R*_ct_*vs.* Pd loading plot of Pd SAs/C_3_N_4_ samples.

To better examine the origin of high activity of our SAs on C_3_N_4,_ we evaluated the charge transfer properties of the Pd-decorated C_3_N_4_ photocatalysts by electrochemical impedance spectroscopy (EIS). The measurements were performed in the 0.1 M Na_2_SO_4_ electrolyte at −0.5 V *vs.* Ag/AgCl, *i.e.*, close to flat band conditions (details are outlined in the ESI-Experimental section†). Fig. 4a presents the Nyquist plots for bare g-C_3_N_4,_ and g-C_3_N_4_ decorated with varying amounts of Pd SAs, and g-C_3_N_4_ decorated with Pd NPs (see the zoomed-in spectra shown in Fig. S12†). The Nyquist plots were fitted using the classic Randle's equivalent circuit model (inset of Fig. 4a).^41,42^ The significantly smaller radius of the fitted curve for Pd SAs/C_3_N_4_, compared to bare C_3_N_4_, indicates a substantial reduction in charge transfer resistance (*R*_ct_) upon Pd SA loading. Quantitative fitting data in Table S4† show a 98-fold decrease in *R*_ct_ due to Pd SA incorporation (already at a concentration of 0.05 mM). Fig. 4b shows the *R*_ct_ values plotted against Pd SA loading, showing that even a minimal amount of Pd SAs (0.03 wt%) can dramatically enhance the charge transfer of C_3_N_4_ to the electrolyte. This aligns with the low loading required to achieve peak efficiency in photocatalytic H_2_ production. Conversely, Pd NPs on C_3_N_4_ also reduce *R*_ct_ compared to bare C_3_N_4_ (Fig. 4b) but require a much higher loading (1.5 wt%) to achieve a similar reduction in charge transfer resistance, as compared to the Pd SA-loaded sample, which achieves this with just 0.03 wt%. PEIS measurements for all the samples were measured using a 365 nm LED (as described in the ESI Experimental section†). As shown in Fig. S13,† the results indicated a similar trend to the EIS data collected in the dark (Fig. 4 and Table S4†), although *R*_ct_ values were different. Under illumination, *R*_ct_ values decreased due to enhanced charge transfer dynamics in the presence of light (Fig. S13 and Table S5†). Notably, Pd SAs demonstrated lower *R*_ct_ values compared to Pd nanoparticles, indicating the superior performance of Pd SAs. These results underscore the effectiveness of small Pd SA quantities in significantly improving the charge transfer characteristics of C_3_N_4_.

Incident photon-to-current conversion efficiency (IPCE) measurements were also conducted to assess the photoelectrochemical characteristics of C_3_N_4_ and Pd SAs/C_3_N_4_. Fig. S14† shows photocurrent spectra for both samples with a photocurrent onset in the visible range. The bandgap (determined from a replot of the photocurrent data according to an indirect transition, Fig. S14-inset†) was 2.7 eV, consistent with the typical bandgap of g-C_3_N_4_.^14,43^ Additionally, the photocurrent (Fig. S15†) increases after the decoration of Pd SAs on g-C_3_N_4_ (under near flat band conditions), which confirms the beneficial effect of Pd SAs in promoting charge transfer under illumination conditions.

The enhanced performance of Pd-SAs deposited by our decoration technique, compared with the literature, must be attributed to the used precursor Pd(NH_3_)_4_^2+^ that leads to the direct formation of active Pd–N configurations (as confirmed by EXAFS, Fig. 2e and f) – in the literature, such sites are regarded as premier active sites in C_3_N_4._^16,20,44,45^

The presence of NH_3_ groups in Pd(NH_3_)_4_^2+^ ions may promote the formation of such a Pd–N coordination structure compared to the chloride-coordinated Pd precursors due to suitable ligand exchange energetics.^46,47^ Furthermore, the Pd^2+^ in Pd(NH_3_)_4_Cl_2_ enables stronger interactions with nitrogen atoms in C_3_N_4_ than the Pd^4+^ in (NH_4_)_2_PdCl_6_ or Pd^2+^ in PdCl_2_.^48,49^ Evidently, chloro-coordinated Pd precursors in the Pd^2+^ or Pd^4+^ state are either adsorbed onto the C_3_N_4_ surface at less specific sites, as shown by the XPS Pd 3d spectra (Fig. S1a†) and the significant presence of Cl detected in the XPS Cl 2p spectra (Fig. S1b†), or reduced and form metallic Pd agglomerates, as clearly observed in the SEM images (Fig. S2†). The tetraammonium complex, on the other hand, leads without any evident change in the reduction state to active Pd^*δ*+^ (*δ* ≈ 2) N-coordinated SAs, accompanied by the complete loss of Cl coordination during the reaction (Fig. S8†). Therefore, Pd(NH_3_)_4_Cl_2_ is identified as the most suitable precursor for direct reactive deposition of Pd SAs on C_3_N_4_.

Considering that many literature studies, particularly those involving DFT calculations, suggest that Pd in an N_4_-coordination on C_3_N_4_ exhibits the highest degree of stability and activity, one may conclude that Pd²⁺ undergoes a ligand exchange process to form this active Pd–N_4_ configuration. This means that the process is of self-homing nature (as described for the reactive deposition of Pt)^23–25,35^*i.e.*, the Pd precursor reacts and deposits Pd SAs at most active surface sites on g-C_3_N_4_, these sites then provide a maximized electron transfer and thus are highly catalytically active. This explains why such a low loading of Pd SAs is sufficient in our work to achieve maximised photocatalytic H_2_ production efficiency compared to the higher Pd loadings required, as reported in most literature studies (Table S3†).^14,45,49,50^

The remarkable activity becomes particularly clear if the present data are compared to the work of Akinaga *et al.*,^14^ where a 0.5 wt% Pd SA loading was required to maximize the photocatalytic H_2_ production activity, *i.e.*, our very low Pd SA loading of 0.05 wt% demonstrates a tenfold increase in efficiency. This superior performance highlights the importance of the attachment chemistry and process of Pd SAs within C_3_N_4,_*i.e.*, processes that lead to Pd SAs located at the most active sites can lead to maximized efficiency with minimal Pd usage, avoiding the waste of Pd associated with random Pd SA or NP deposition.

## Conclusion

In this work, we successfully integrated Pd SAs onto/into exfoliated g-C_3_N_4_*via* a reactive deposition method, achieving a controllable uniform loading of highly active Pd SAs in a Pd–N configuration on g-C_3_N_4_. Notably, using reactive deposition from a Pd(NH_3_)_4_Cl_2_ precursor a low loading of 0.05 wt% Pd SAs on C_3_N_4_ with a density of 1.6 × 10^6^ μm^−2^ can achieve a maximum H_2_ production rate of 0.24 mmol h^−1^ mg^−1^ Pd, significantly higher than that of Pd nanoparticles decorated on g-C_3_N_4_ and also ten times higher than that of Pd SAs decorated on g-C_3_N_4_ using other reported approaches. Other tested precursors may also deliver SA attachment but lack the high co-catalytic activity. These results underline the importance of the attachment mechanism in creating a SA/substrate coupling with minimized charge transfer resistance and thus maximized co-catalytic activity – in a most effective way, the process is self-homing, *i.e.*, activation takes place where it is most effective.

## Author contributions

Velu Jeyalakshmi conducted the majority of the experimental work, including synthesis, characterization, data analysis, and drafting of the manuscript. Siming Wu contributed significantly to data analysis and revision of the original draft. Shanshan Qin participated in data analysis, while Xin Zhou performed HAADF-STEM analysis. Bidyut Bikash Sarma and Dimitry E. Doronkin conducted XAS and EXAFS analyses and Jan Kolařík carried out AAS analysis. Miroslav Šoóš supervised the experiments and managed funding acquisition. Patrik Schmuki conceived the idea, supervised the experiments, analyzed the data, revised the manuscript, and secured funding. All authors contributed to the preparation of the manuscript and approved the final manuscript.

## Conflicts of interest

There are no conflicts to declare.

## Supplementary Material

SC-016-D4SC08589B-s001

## Data Availability

The data that support the findings of this study are available within the article and ESI.†

## References

[cit1] Li Y., Tsang S. C. E. (2020). Recent progress and strategies for enhancing photocatalytic water splitting. Mater. Today Sustain..

[cit2] Nishioka S., Osterloh F. E., Wang X., Mallouk T. E., Maeda K. (2023). Photocatalytic water splitting. Nat. Rev. Methods Primers.

[cit3] Nakata K., Fujishima A. (2012). TiO2 photocatalysis: design and applications. J. Photochem. Photobiol., C.

[cit4] Roy P., Berger S., Schmuki P. (2011). TiO2 nanotubes: synthesis and applications. Angew. Chem., Int. Ed..

[cit5] Peiris S., de Silva H. B., Ranasinghe K. N., Bandara S. V., Perera I. R. (2021). Recent development and future prospects of TiO2 photocatalysis. J. Chin. Chem. Soc..

[cit6] Hernández-Alonso M. D., Fresno F., Suárez S., Coronado J. M. (2009). Development of alternative photocatalysts to TiO 2: challenges and opportunities. Energy Environ. Sci..

[cit7] Bhanderi D., Lakhani P., Modi C. K. (2024). Graphitic carbon nitride (g-C3N4) as an emerging photocatalyst for sustainable environmental applications: a comprehensive review. RSC sustain..

[cit8] Wang Q., Li Y., Huang F., Song S., Ai G., Xin X., Zhao B., Zheng Y., Zhang Z. (2023). Recent advances in g-C3N4-based materials and their application in energy and environmental sustainability. Molecules.

[cit9] Pei J., Li H., Zhuang S., Zhang D., Yu D. (2023). Recent Advances in g-C3N4 Photocatalysts: A Review of Reaction Parameters, Structure Design and Exfoliation Methods. Catalysts.

[cit10] Zheng Y., Jiao Y., Jaroniec M., Qiao S. Z. (2015). Advancing the electrochemistry of the hydrogen-evolution reaction through combining experiment and theory. Angew. Chem., Int. Ed..

[cit11] Gao C., Low J., Long R., Kong T., Zhu J., Xiong Y. (2020). Heterogeneous single-atom photocatalysts: fundamentals and applications. Chem. Rev..

[cit12] Xia Y., Sayed M., Zhang L., Cheng B., Yu J. (2021). Single-atom heterogeneous photocatalysts. Chem Catal..

[cit13] Kerketta U., Tesler A. B., Schmuki P. (2022). Single-atom Co-catalysts employed in titanium dioxide photocatalysis. Catalysts.

[cit14] Akinaga Y., Kawawaki T., Kameko H., Yamazaki Y., Yamazaki K., Nakayasu Y., Kato K., Tanaka Y., Hanindriyo A. T., Takagi M. (2023). Metal Single-Atom Cocatalyst on Carbon Nitride for the Photocatalytic Hydrogen Evolution Reaction: Effects of Metal Species. Adv. Funct. Mater..

[cit15] Jia X., Zhao J., Zhang W., Fu X., Long J., Gu Q., Gao Z. (2022). Single-Atomic Pd Embedded 2D g-C3N4 Homogeneous Catalyst Analogues for Efficient LMCT Induced Full-Visible-Light Photocatalytic Suzuki Coupling. ChemistrySelect.

[cit16] Hu F., Leng L., Zhang M., Chen W., Yu Y., Wang J., Horton J. H., Li Z. (2020). Direct synthesis of atomically dispersed palladium atoms supported on graphitic carbon nitride for efficient selective hydrogenation reactions. ACS Appl. Mater. Interfaces.

[cit17] Chen Z., Mitchell S., Vorobyeva E., Leary R. K., Hauert R., Furnival T., Ramasse Q. M., Thomas J. M., Midgley P.
A., Dontsova D. (2017). Stabilization of single metal atoms on graphitic carbon nitride. Adv. Funct. Mater..

[cit18] Xu R., Xu B., You X., Shao D., Gao G., Li F., Wang X.-L., Yao Y.-F. (2023). Preparation of single-atom palladium catalysts with high photocatalytic hydrogen production performance by means of photochemical reactions conducted with frozen precursor solutions. J. Mater. Chem. A.

[cit19] Ren M., Zhang X., Liu Y., Yang G., Qin L., Meng J., Guo Y., Yang Y. (2022). Interlayer palladium-single-atom-coordinated cyano-group-rich graphitic carbon nitride for enhanced photocatalytic hydrogen production performance. ACS Catal..

[cit20] Cao S., Li H., Tong T., Chen H. C., Yu A., Yu J., Chen H. M. (2018). Single-atom engineering of directional charge transfer channels and active sites for photocatalytic hydrogen evolution. Adv. Funct. Mater..

[cit21] Suja P., John J., Rajan T., Anilkumar G. M., Yamaguchi T., Pillai S. C., Hareesh U. (2023). Graphitic carbon nitride (gC 3 N 4) based heterogeneous single atom catalysts: synthesis, characterisation and catalytic applications. J. Mater. Chem. A.

[cit22] Li S., Kan Z., Wang H., Bai J., Liu Y., Liu S., Wu Y. (2023). Single-atom photo-catalysts: synthesis, characterization, and applications. Nano Mater. Sci..

[cit23] Wu S. M., Schmuki P. (2024). Single Atom Cocatalysts in Photocatalysis. Adv. Mater..

[cit24] Wang Y., Qin S., Denisov N., Kim H., Bad'ura Z., Sarma B. B., Schmuki P. (2023). Reactive Deposition Versus Strong Electrostatic Adsorption (SEA): A Key to Highly Active Single Atom Co-Catalysts in Photocatalytic H2 Generation. Adv. Mater..

[cit25] Qin S., Will J., Kim H., Denisov N., Carl S., Spiecker E., Schmuki P. (2023). Single atoms in photocatalysis: low loading is good enough. ACS Energy Lett..

[cit26] Cha G., Mazare A., Hwang I., Denisov N., Will J., Yokosawa T., Badura Z., Zoppellaro G., Tesler A. B., Spiecker E. (2022). A facile “dark”-deposition approach for Pt single-atom trapping on facetted anatase TiO2 nanoflakes and use in photocatalytic H2 generation. Electrochim. Acta.

[cit27] Wu Z., Hwang I., Cha G., Qin S., Tomanec O., Badura Z., Kment S., Zboril R., Schmuki P. (2022). Optimized Pt single atom harvesting on TiO2 nanotubes—Towards a most efficient photocatalyst. Small.

[cit28] Torres-Pinto A., Sampaio M. J., Silva C. G., Faria J. L., Silva A. M. (2019). Metal-free carbon nitride photocatalysis with in situ hydrogen peroxide generation for the degradation of aromatic compounds. Appl. Catal., B.

[cit29] Marchal C., Cottineau T., Méndez-Medrano M. G., Colbeau-Justin C., Caps V., Keller V. (2018). Au/TiO2–gC3N4 nanocomposites for enhanced photocatalytic H2 production from water under visible light irradiation with very low quantities of sacrificial agents. Adv. Energy Mater..

[cit30] Karimi-Nazarabad M., Ahmadzadeh H., Goharshadi E. K. (2021). Porous perovskite-lanthanum cobaltite as an efficient cocatalyst in photoelectrocatalytic water oxidation by bismuth doped g-C3N4. Sol. Energy.

[cit31] Fina F., Callear S. K., Carins G. M., Irvine J. T. (2015). Structural investigation of graphitic carbon nitride via XRD and neutron diffraction. Chem. Mater..

[cit32] Wang N., Wang J., Hu J., Lu X., Sun J., Shi F., Liu Z.-H., Lei Z., Jiang R. (2018). Design of palladium-doped g-C3N4 for enhanced photocatalytic activity toward hydrogen evolution reaction. ACS Appl. Energy Mater..

[cit33] Li L., Dai X., Lu M., Guo C., Wabaidur S. M., Wu X.-L., Lou Z., Zhong Y., Hu Y. (2024). Electron-enriched single-Pd-sites on g-C3N4 nanosheets achieved by in-situ anchoring twinned Pd nanoparticles for efficient CO2 photoreduction. Adv. Powder Mater..

[cit34] Mondal S., Sahoo L., Banoo M., Vaishnav Y., Prabhakaran Vinod C., Gautam U. K. (2024). Enhancing the Catalytic Activity of Pd Nanocrystals towards Suzuki Cross-Coupling by g-C3N4 Photosensitization. ChemNanoMat.

[cit35] Wu S.-M., Wu L., Denisov N., Badura Z., Zoppellaro G., Yang X.-Y., Schmuki P. (2024). Pt Single Atoms on TiO2 Can Catalyze Water Oxidation in Photoelectrochemical Experiments. J. Am. Chem. Soc..

[cit36] Liu P., Huang Z., Gao X., Hong X., Zhu J., Wang G., Wu Y., Zeng J., Zheng X. (2022). Synergy between palladium single atoms and nanoparticles via hydrogen spillover for enhancing CO2 photoreduction to CH4. Adv. Mater..

[cit37] Ho P. H., Woo J.-W., Ilmasani R. F., Han J., Olsson L. (2021). The role of Pd–Pt interactions in the oxidation and sulfur resistance of bimetallic Pd–Pt/γ-Al2O3 diesel oxidation catalysts. Ind. Eng. Chem. Res..

[cit38] Wang M., Shen S., Li L., Tang Z., Yang J. (2017). Effects of sacrificial reagents on photocatalytic hydrogen evolution over different photocatalysts. J. Mater. Sci..

[cit39] Kumaravel V., Imam M. D., Badreldin A., Chava R. K., Do J. Y., Kang M., Abdel-Wahab A. (2019). Photocatalytic hydrogen production: role of sacrificial reagents on the activity of oxide, carbon, and sulfide catalysts. Catalysts.

[cit40] Denisov N., Qin S., Will J., Vasiljevic B. N., Skorodumova N. V., Pašti I. A., Sarma B. B., Osuagwu B., Yokosawa T., Voss J. (2023). Light-Induced Agglomeration of Single-Atom Platinum in Photocatalysis. Adv. Mater..

[cit41] Randles J. E. B. (1947). Kinetics of rapid electrode reactions. Discuss. Faraday Soc..

[cit42] Wang Z., Murphy A., O'Riordan A., O'Connell I. (2021). Equivalent impedance models for electrochemical nanosensor-based integrated system design. Sensors.

[cit43] Wang X., Maeda K., Thomas A., Takanabe K., Xin G., Carlsson J. M., Domen K., Antonietti M. (2009). A metal-free polymeric photocatalyst for hydrogen production from water under visible light. Nat. Mater..

[cit44] Vilé G., Albani D., Nachtegaal M., Chen Z., Dontsova D., Antonietti M., López N., Pérez-Ramírez J. (2015). Ein stabiler “Single-site”-Palladiumkatalysator für Hydrierungen. Angew. Chem..

[cit45] Liu L., Wu X., Wang L., Xu X., Gan L., Si Z., Li J., Zhang Q., Liu Y., Zhao Y., Ran R. (2019). Atomic palladium on graphitic
carbon nitride as a hydrogen evolution catalyst under visible light irradiation. Commun. Chem..

[cit46] Ryabov A., Kazankov G., Yatsimirskii A., Kuz'mina L., Burtseva O. Y., Dvortsova N., Polyakov V. (1992). Synthesis by ligand exchange, structural characterization, and aqueous chemistry of ortho-palladated oximes. Inorg. Chem..

[cit47] Maitlis P. , Metal Complexes: The Organic Chemistry of Palladium, Elsevier, 2012, pp. 1–103

[cit48] Pazderski L. (2008). 15N NMR coordination shifts in Pd (II), Pt (II), Au (III), Co (III), Rh (III), Ir (III), Pd (IV), and Pt (IV) complexes with pyridine, 2, 2′-bipyridine, 1, 10-phenanthroline, quinoline, isoquinoline, 2, 2′-biquinoline, 2, 2′: 6′, 2′-terpyridine and their alkyl or aryl derivatives. Magn. Reson. Chem..

[cit49] Wang N., Wang J., Hu J., Lu X., Sun J., Shi F., Liu Z.-H., Lei Z., Jiang R. (2018). Design of Palladium-Doped g-C 3 N 4 for Enhanced Photocatalytic Activity toward Hydrogen Evolution Reaction. ACS Appl. Energy Mater..

[cit50] Xu R., Xu B., You X., Shao D., Gao G., Li F., Wang X.-L., Yao Y.-F. (2023). Preparation of single-atom palladium catalysts with high photocatalytic hydrogen production performance by means of photochemical reactions conducted with frozen precursor solutions. J. Mater. Chem. A.

