# Supplementary material for: Pd single atoms on g-C3N4 photocatalysts: minimum loading for maximum activity
Source: Chem Sci. 2025 Feb 12;16(11):4788–95. doi: 10.1039/d4sc08589b (PMC11816733; doi:10.1039/d4sc08589b)
Supplement: SC-016-D4SC08589B-s001 [file SC-016-D4SC08589B-s001.pdf]

Supplementary Information (SI) for Chemical Science.
This journal is © The Royal Society of Chemistry 2025

Supporting information

Pd Single atoms on g-C_3N_4 photocatalysts: Minimum loading for a maximum activity

Velu Jeyalakshmi,[1,2#] Siming Wu,[1#] Shanshan Qin,[1] Xin Zhou,[1] Bidyut Bikash Sarma,[3] Dimitry E. Doronkin,[4] Jan Kolařík,[5] Miroslav Šoóš,[2] and Patrik Schmuki[1,5]

[1]Department of Materials Science WW4-LKO, Friedrich-Alexander-University of Erlangen-Nuremberg, Martensstrasse 7, 91058 Erlangen, Germany.

[2]Department of Chemical Engineering, University of Chemistry and Technology, Technická 3, Prague 160 00, Czech Republic.

[3]Laboratoire de Chimie de Coordination (LCC), CNRS, Université de Toulouse, INPT, UPR 8241, 205 route de Narbonne, 31077 Toulouse Cedex 4, France.

[4]Institute of Catalysis Research and Technology, KIT, Hermann-von Helmholtz Platz 1, 76344 Eggenstein-Leopoldshafen, Germany.

[5]Regional Centre of Advanced Technologies and Materials, Šlechtitelů 27, 78371 Olomouc, Czech Republic

Email: schmuki@ww.uni-erlangen.de

[#]Equal Contribution

Experimental Section:

Materials: Dicyandiamide ($C_2H_4N_4$, Alfa Aesar, 99%), Melamine ($C_3H_6N_6$, Alfa Aesar, 99%), Tetraamine Palladium (II) chloride ($Pd(NH_3)_4Cl_2$. xH_2O, Alfa Aesar), Ammonium hexachloro palladate (IV)-[$(NH_4)_2[PdCl_6]$, Sigma Aldrich], Palladium chloride [$PdCl_2$- Sigma Aldrich], Triethanolamine ($N(CH_2CH_2OH)_3$, Sigma-Aldrich, ≥99%), Sodium sulphate (Na_2SO_4, Sigma-Aldrich, ≥99%), ultra-pure water used throughout all the experiments.

Synthesis of graphitic carbon nitride (g-C_3N_4)

Thermal polycondensation followed by thermal exfoliation is employed for the synthesis of g-C_3N_4.[1-3] An equimolar mixture of melamine and dicyandiamide is thoroughly ground in an agate mortar to ensure homogeneity. The resulting ground powder is placed in an alumina crucible with a lid and calcined in static air at 530 °C for 2 hours in a muffle furnace with a heating rate of 5 °C/min. Subsequently, the calcined powders washed with ultra-pure distilled water, filtered and dried overnight at 70 °C. The obtained powder is then placed in an open crucible and calcined in static air at 500 °C for 2 hours, resulting in the final product, denoted as C_3N_4.

Synthesis of Single atom Pd SAs/C_3N_4

Using a "reactive deposition" approach[4, 5] Pd SAs/C_3N_4 is synthesised within a controlled environment. A suspension of 60 mg of C_3N_4 and 30 mL of ultrapure water is placed in a sealed quartz cell then subjected to Argon purging for 20 minutes to eliminate residual oxygen. Subsequently, varying concentrations of Tetra amine palladium (II) chloride (10 mM, 2 mM, 0.5 mM, 0.05 mM, 0.005 mM, 0.002 mM and 0.0005 mM) was added. The system is then sealed and kept in darkness for one hour under continuous stirring. Following the dark deposition process, the resulting powder is thoroughly washed with deionized (DI) water via centrifugation, dried at 70 °C overnight and denoted as Pd SAs/C_3N_4.

This procedure is also repeated with alternative palladium precursors, including Ammonium hexachloro palladate (IV) and palladium chloride, using a 2 mM concentration.

Synthesis of Pd NPs/C_3N_4

Pd nanoparticles are synthesized via the photo deposition method and are used as a reference sample.[6, 7] A 3 wt% palladium (Pd) from Tetraamine palladium (II) chloride precursor is added to a suspension containing 60 mg of C_3N_4 and 30 mL of 10 vol% tri-ethanol amine solution. The solution is then exposed to 3*365 nm LED irradiation (65 $mWcm^{-2}$) under continuous stirring and N_2 purging for 6 hours. Following irradiation, the sample is thoroughly washed with DI water through centrifugation and dried overnight at 70 °C. The resultant powder is labelled as "Pd NPs".

Photocatalytic H_2 evolution

The photocatalytic hydrogen evolution experiments are conducted in a sealed quartz reactor.[3] 10 mg of the synthesized photocatalyst is dispersed within 10 mL of 10 vol% Triethanolamine solution, followed by a 15 minutes purge with Argon to eliminate residual oxygen from the solution. Subsequently, completely sealed system is irradiated using 365 nm LED lamp with a power density of 65 $mWcm^{-2}$ for 3 hours. The evolved hydrogen is analysed using gas chromatography (GCMS-QO2010SE, SHIMADZU) equipped with a thermal conductivity detector (TCD) at consistent intervals of one hour.

Characterization

The morphology and elemental composition of the photocatalysts underwent analysis using advanced equipment. Field-emission scanning electron microscope (FESEM, Hitachi S-4800) equipped with energy dispersive X-ray spectroscopy (EDAX Genesis) was utilized for morphological assessment. High angle annular dark-field scanning transmission electron microscopy (HAADF-STEM) imaging was performed employing an aberration corrected Thermo Fisher Scientific Spectra 200 operating at 200 keV, and featuring an ultra-high brightness cold field emission gun (X-CFEG). Structural examination was carried out using X'pert Philips MPD diffractometer with a Panalytical X'celerator detector, utilizing a Cu Kα radiation source (λ = 1.54056 Å). Additionally, X-ray photoelectron spectroscopy (XPS, PHI 5600) was employed to scrutinize oxidation states and chemical composition with all XPS spectra referenced to a standard C1s peak position of 288.1 eV. Diffuse reflectance infra-red spectroscopy (DRIFTS): In-situ CO-DRIFTS experiments were conducted on a VERTEX 70

FTIR spectrometer from Bruker equipped with a Mercury-Cadmium Telluride (MCT) detector. A Harrick Praying Mantis optics and the Harrick high temperature cell with a flat CaF_2 window were used. The samples were measured in powder form. All the catalysts were pre-treated by purging Ar and heating to 353 K for 1 h and then the spectra were collected under 1% CO/Ar for 1h in reflectance mode between 1000-4000 cm^{-1}. A 4 cm^{-1} spectral resolution was used for all measurements. The spectra are reported in logarithm log(1/R) or in Kubelka-Munk. For the background spectra, 150 scans were collected and averaged under Ar flow and for the samples 150 scans were collected per measurement. X-ray Absorption Spectroscopy (XAS): X-ray absorption spectra at the Pd K-edge (24350 eV) were collected at the P65 beamline of the PETRA III synchrotron source at Deutsches Elektronen-Synchrotron (DESY), Hamburg, Germany. The incident energies were scanned with a Si (311) double crystal monochromator (DCM). For measuring the reference samples, pellets diluted with cellulose were used, and the measurements were conducted in transmission. For ex-situ measurements, the catalysts were kept inside a plastic sample holder without dilution and the measurements were conducted in fluorescence mode using a Hitachi Vortex-ME4 silicon drift detector. The processing of the XAS results (data reduction, alignment, normalization) has been performed using the Athena code (version 0.9.26).

Photoelectrochemical studies

Photoelectrochemical and electrochemical characteristics of the materials were meticulously analysed employing various methods including Incident Photon-to-Current Conversion Efficiency (IPCE), transient photocurrent studies and electrochemical impedance spectroscopy (EIS). IPCE spectra, covering the 300-800 nm wavelength range, were captured using a 150 W Xenon arc lamp in conjunction with a Cornerstone motorized 1/8 m monochromator from LOT-Oriel Instruments at 0 V (vs. Ag/AgCl). The EIS measurements were conducted using a Zahner IM6 electrochemical workstation (Zahner Elektrik, Kronach, Germany), with impedance response recorded in the dark at -0.5 V (vs. Ag/AgCl) with a 5mV potential perturbation. PEIS measurements were conducted using the same parameter as dark conditions, but in the presence of 365 nm LED. Data analyses were executed using the “EIS Spectrum Analyzer” software. Additionally, the transient photocurrent studies were conducted with a 0.5 V applied potential (vs. Ag/AgCl) using a 365 nm LED in a 60 second on-off light cycle.

All the measurements were performed in a three-electrode cell setup comprising the working electrode (synthesized photocatalyst), Pt foil counter electrode, and an Ag/AgCl reference electrode. 0.1 M Na_2SO_4 solution in deionized water was used as electrolyte. The IPCE and transient photocurrent measurements utilized a 0.1 M Na_2SO_4 solution (10 vol.% Triethanolamine in H_2O). The fabrication of working electrode involved dispersing 1 mg of photocatalysts in a solution of 20 μL of Nafion, 50 μL of H_2O and 50 μL of ethanol, followed by sonication for 20 minutes and dip coating of 20 μL of the dispersed solution on a carbon electrode.

Supporting Figures

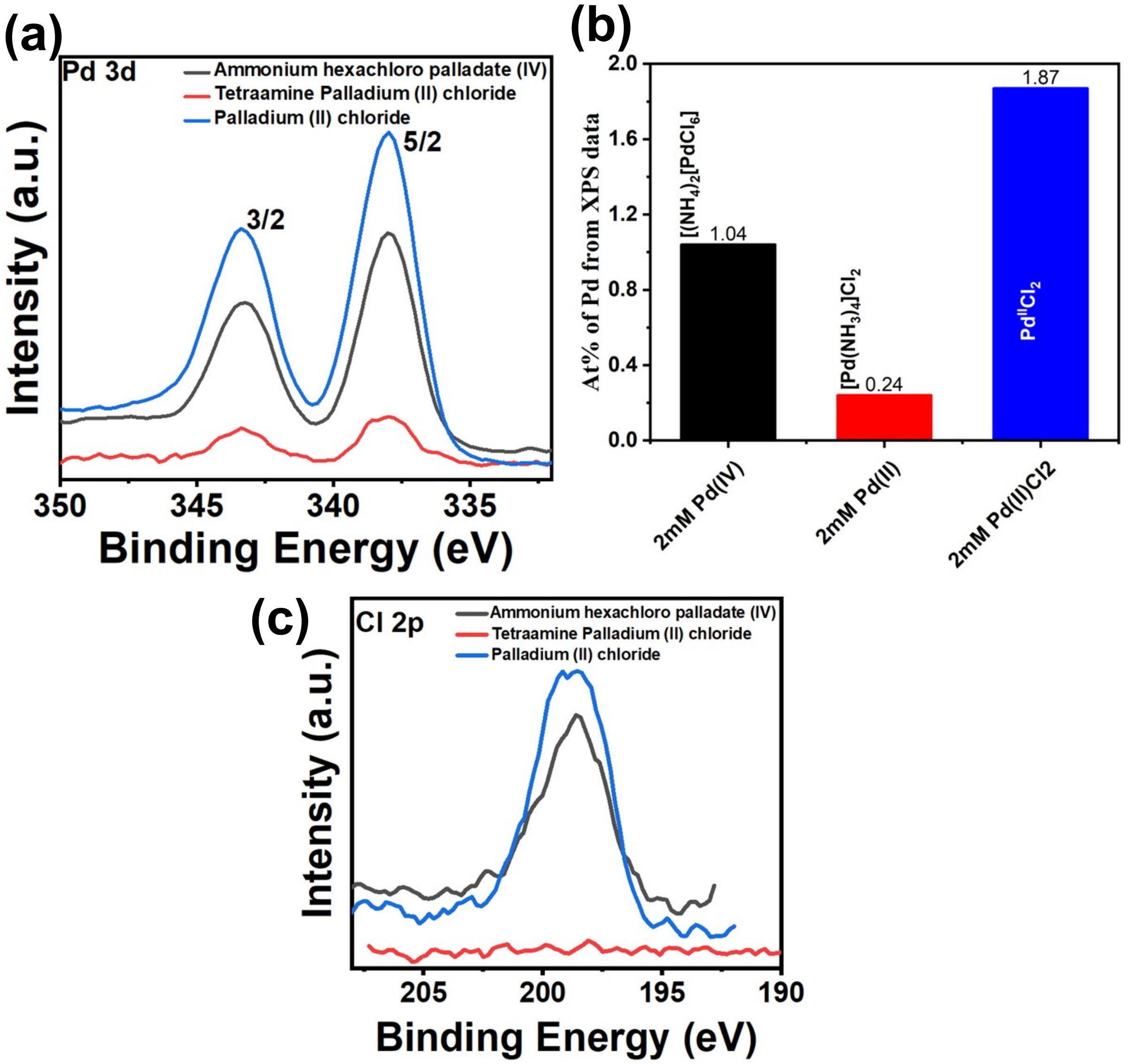

Figure S1 Pd SAs decorated C_3N_4 samples prepared with different precursor's of Pd, i.e. Ammonium hexachloro palladate (IV)-[$(NH_4)_2$ [$PdCl_6$], Tetraamine Palladium (II) chloride $Pd(NH_3)_4Cl_2$, and Palladium chloride [$PdCl_2$] - (a) Pd 3d HR XPS (b) Pd loading derived from the Pd 3d XPS (c) HR XPS of Cl 2p.

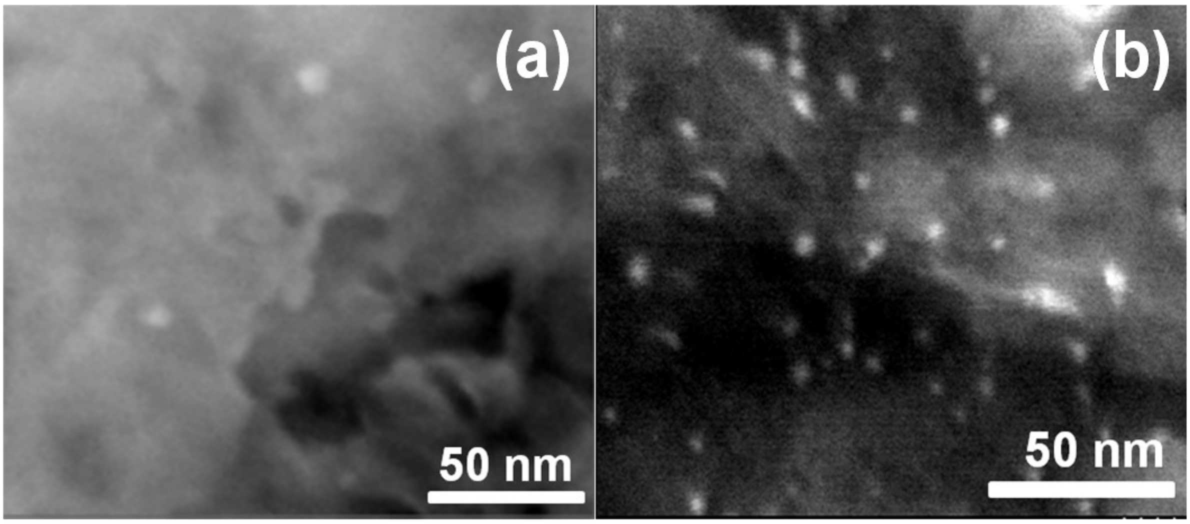

Figure S2 SEM images of Pd deposited on C_3N_4 using different precursors (a) Ammonium hexachloro palladate (IV)-[$(NH_4)_2$ [$PdCl_6$], (b) Palladium chloride-[$PdCl_2$]

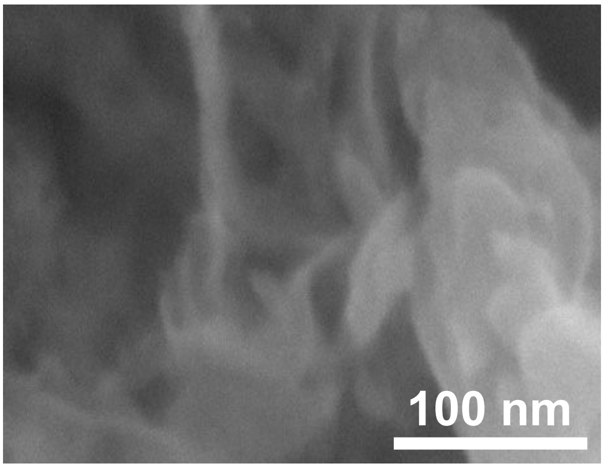

Figure S3 SEM image of C_3N_4

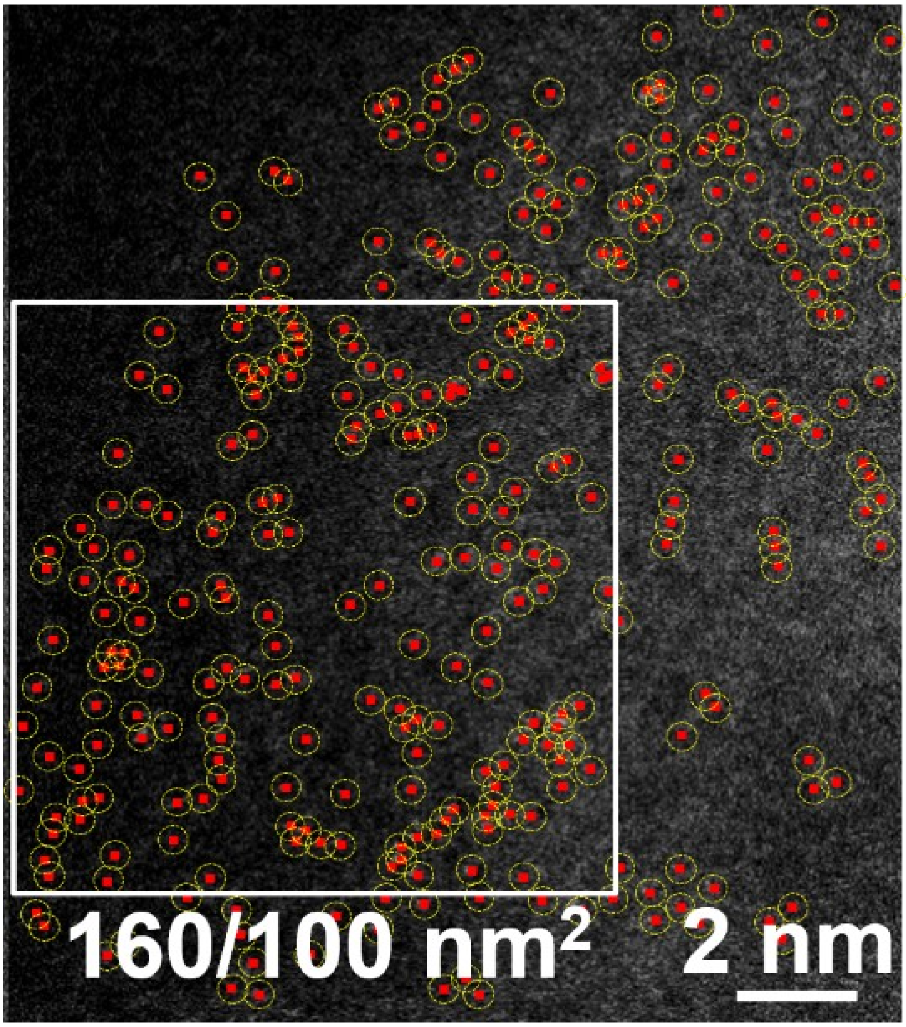

Figure S4 HAADF-STEM image of Pd SAs/C_3N_4 (SA density = 1.6 x10^6 μm^{-2})

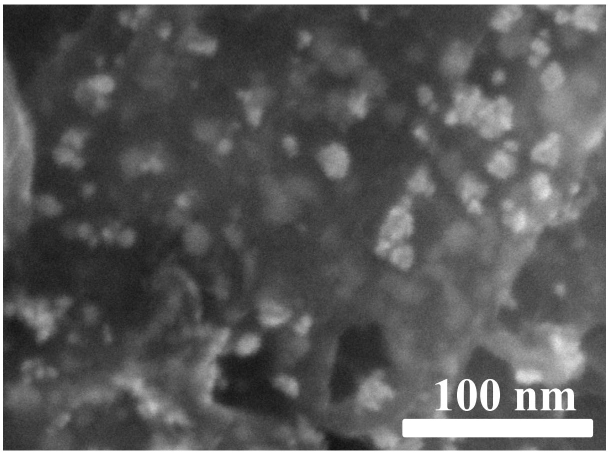

Figure S5 SEM image of Pd NPs decorated C_3N_4 by photo deposition.

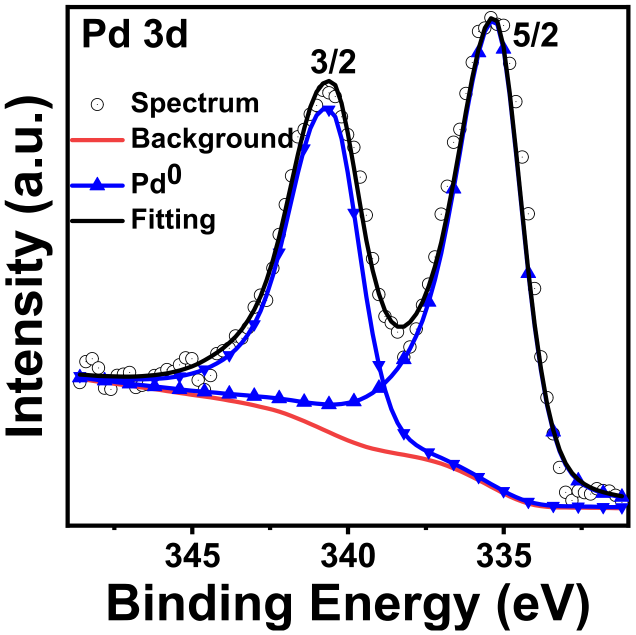

Figure S6 Pd 3d HRXPS spectra for Pd NPs/C_3N_4.

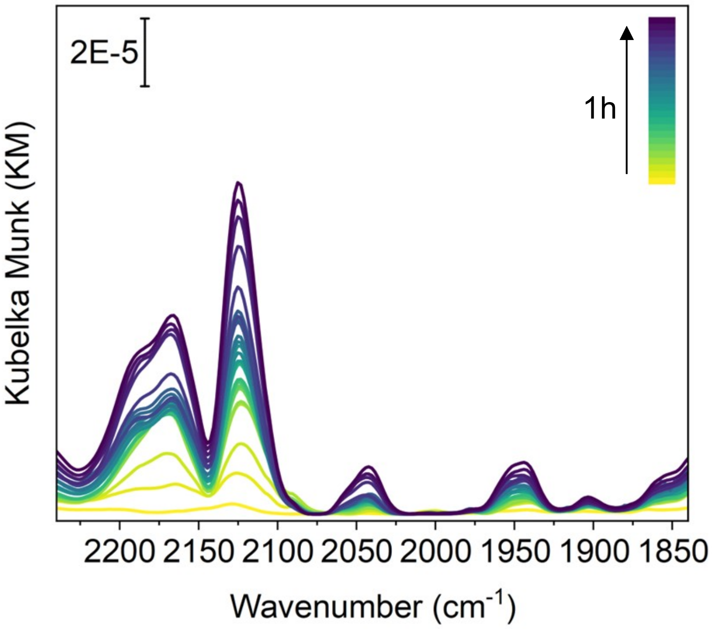

Figure S7 CO-DRIFTS spectra of Pd SAs/C_3N_4. The CO adsorption in the range 2155-2200 cm^{-1} can be ascribed to gas phase CO. Below 2100 cm^{-1}, CO adsorbed over Pd cluster can be observed which formed during CO purging.

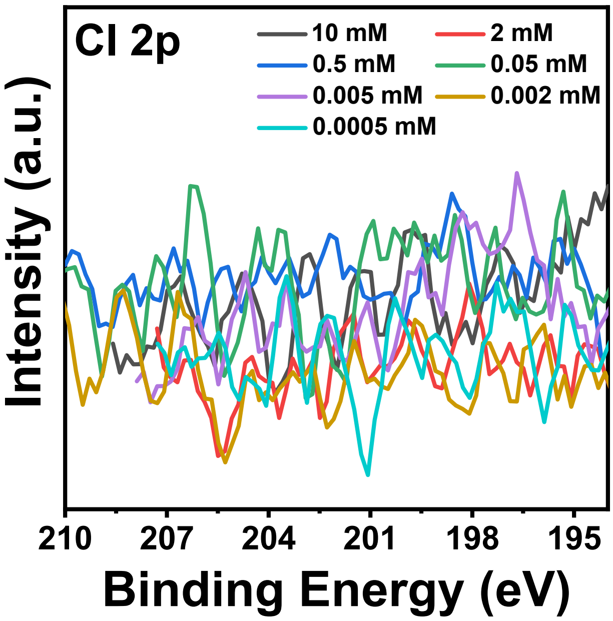

Figure S8 Cl 2p HRXPS spectra for Pd SAs/C_3N_4 with different Pd precursor (Tetraamine palladium (II) chloride) concentration.

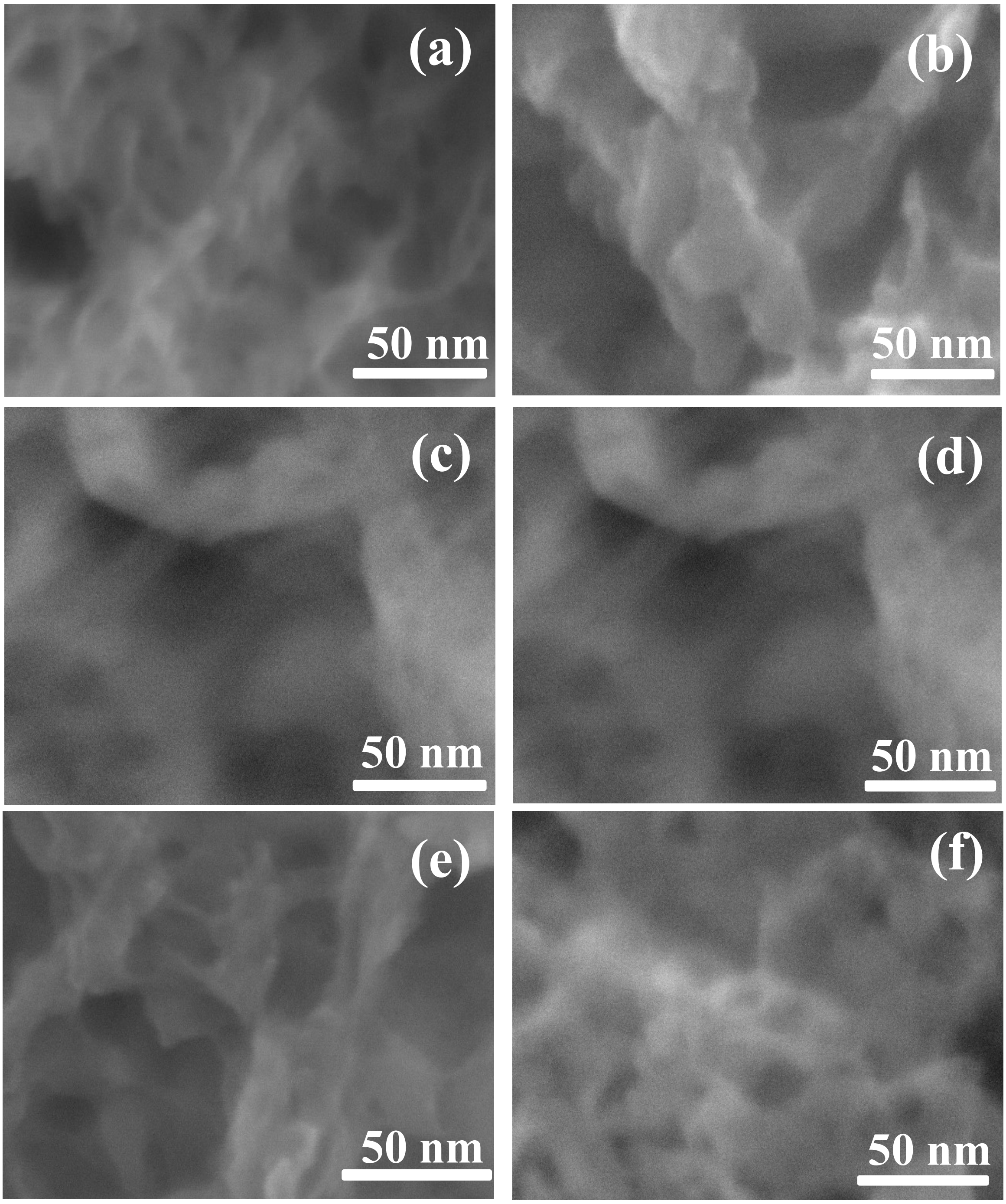

Figure S9 SEM images of Pd SAs/C_3N_4 prepared with (a) 10 mM, (b) 2 mM, (c) 0.5 mM, (d) 0.05 mM, (e) 0.005 mM, (f) 0.0005 mM of Tetraamine palladium (II) chloride.

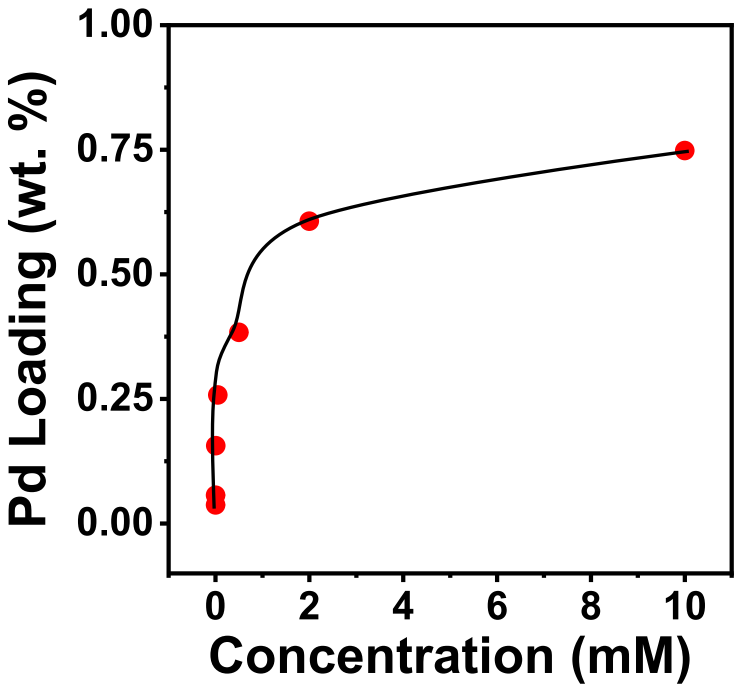

Figure S10 Plot for Pd loading (wt%) vs. Pd precursor concentration used for dark deposition.

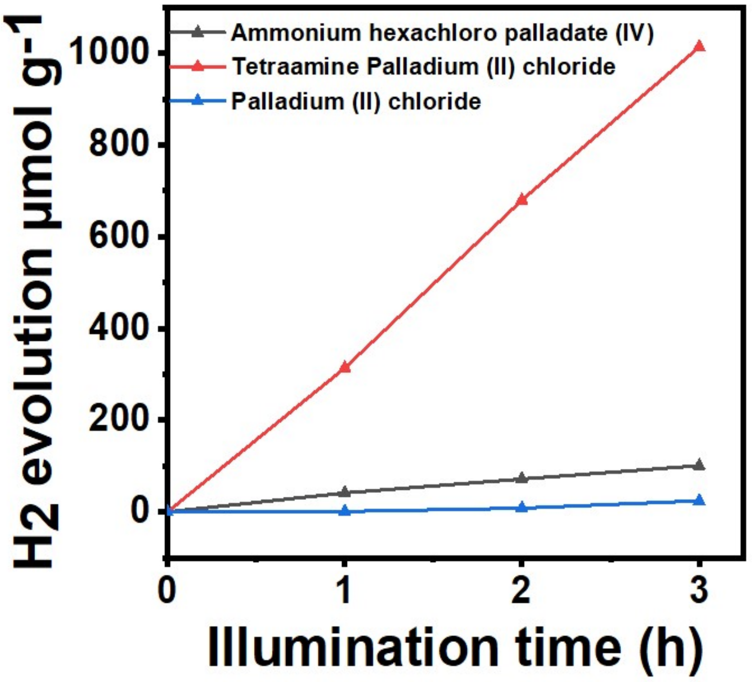

Figure S11 Photocatalytic H_2 evolution from Pd deposited on C_3N_4 using different precursors, i.e. Ammonium hexachloro palladate (IV)-[$(NH_4)_2$ [$PdCl_6$], Tetraamine palladium (II) chloride $Pd(NH_3)_4Cl_2$, and palladium chloride [$PdCl_2$].

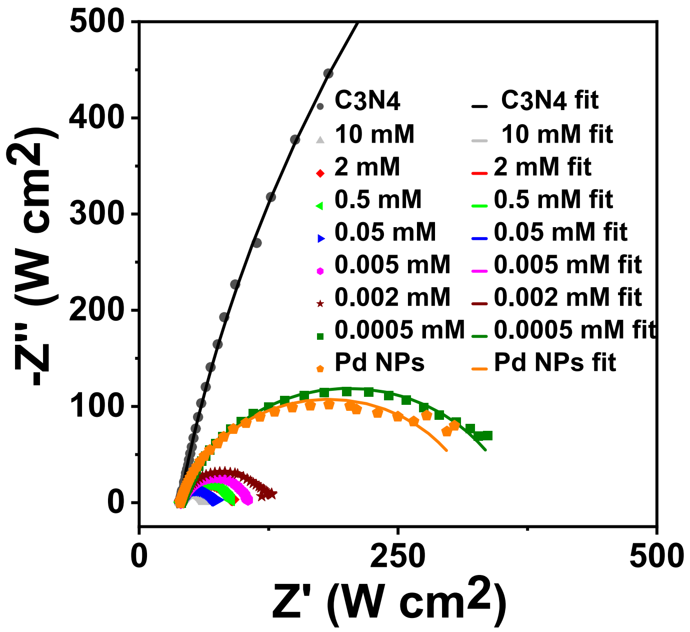

Figure S12 EIS plots of C_3N_4, Pd SAs/C_3N_4 and Pd NPs/C_3N_4 at the voltage -0.5 V (vs Ag/AgCl) in 0.1 M Na_2SO_4 aqueous electrolyte.

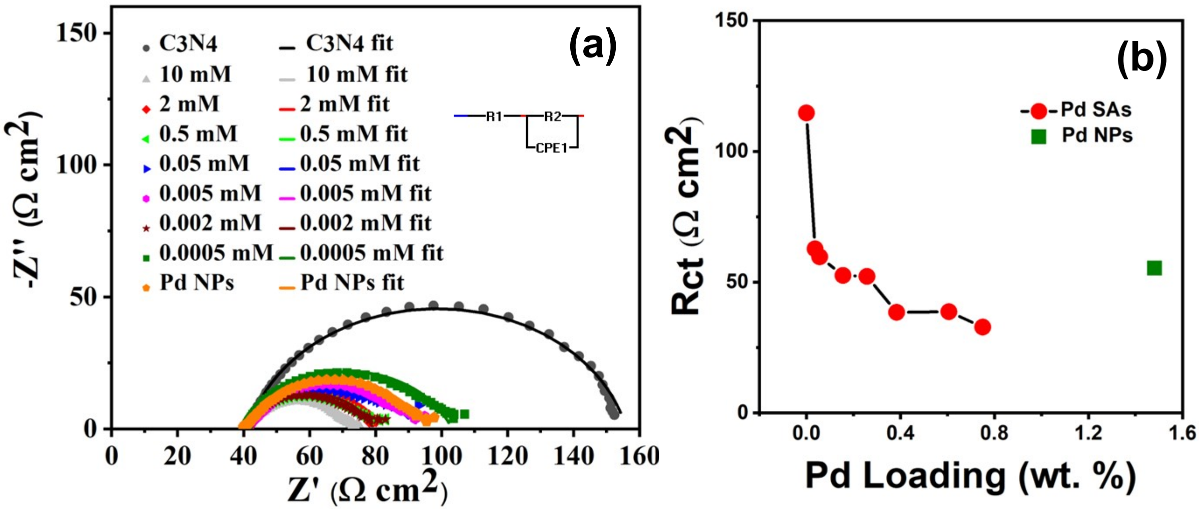

Figure S13 (a) PEIS plots of C_3N_4, Pd SAs/C_3N_4 and Pd NPs/C_3N_4 at the voltage -0.5 V (vs.Ag/AgCl) in 0.1 M Na_2SO_4 aqueous electrolyte under 365 nm LED illumination. The equivalent circuit model used for fitting is depicted in the inset of Figure S13a and (b) Rct vs. Pd loading plot of Pd SAs/C_3N_4 samples.

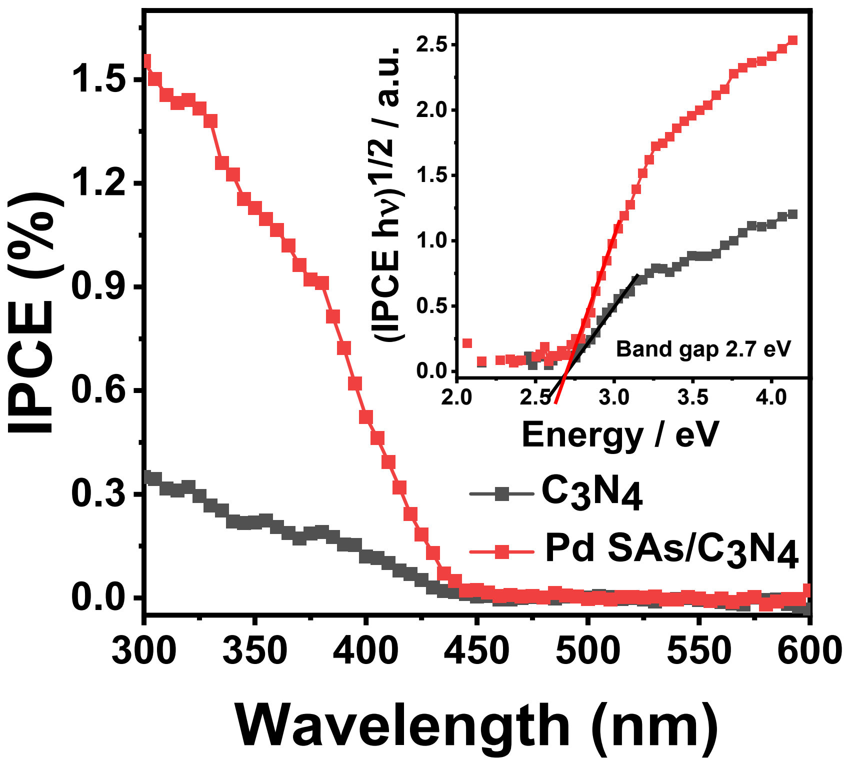

Figure S14 IPCE spectra for C_3N_4 and Pd SAs/C_3N_4. The inset of the band gap from the IPCE spectra.

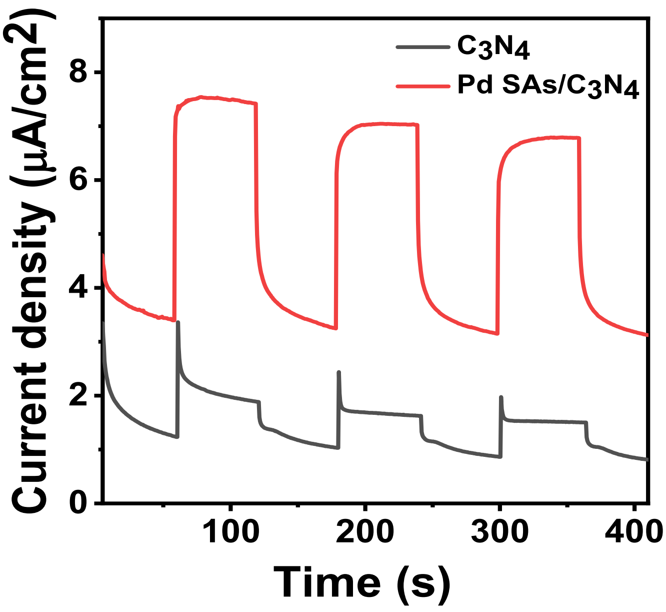

Figure S15 Transient photocurrent for C_3N_4 and Pd SAs/C_3N_4.

Table S1 Weight percent (wt. %) of Pd from AAS measurement

Samples	10 mM	2 mM	0.5 mM	0.05 mM	0.005 mM	0.002 mM	0.0005 mM	Pd NPs
Pd Loading (wt.%)	0.75	0.61	0.38	0.26	0.16	0.05	0.04	1.48

Table S2 Pd atomic loading from XPS spectra

Samples	10 mM	2 mM	0.5 mM	0.05 mM	0.005 mM	0.002 mM	0.0005 mM	Pd NPs
Pd Loading (at%)	0.33	0.24	0.15	0.08	0.02	0.01	0.01	0.46

Table S3 Literature comparison of the photocatalytic H_2 evolution activity over the Pd SAs/C_3N_4

Photocatalyst	Synthesis method	Sacrificial agent	H_2 produced (μmol/h/mg of Pd)	Ref.
0.05 wt% Pd SAs/ C_3N_4	Reactive deposition	TEOA	239	Our work
1.2 wt% Pd/ gC_3N_4	In-situ thermal polymerisation	TEOA	8.3	[8]
0.5 wt% Pd/g-C_3N_4	photo deposition (0.5hr)	MeOH	2.4	[9]
0.1 wt% Pd/g-C_3N_4	liquid-phase adsorption method (18 hrs with Na_2CO_3)	TEOA	0.0009	[10]
0.3 wt% Pd SAs/ g-C_3N_4	Freeze dry	TEOA	8.73	[11]

Table S4 Electrical parameters of the C_3N_4 and Pd SAs/C_3N_4 samples extracted from EIS plots by fitting with an equivalent circuit model

Samples	R_s (Ω cm^2)	R_{ct} (Ω cm^2)	Q_0 (S)	n	C_t (μF cm^{-2})
C_3N_4	39.14	3317.9	0.00043	0.889	457.76
10 mM	40.14	23.3	0.00146	0.816	682.8
2mM	40.29	49.19	0.001	0.839	562.63

0.5 mM	40.07	49.32	0.00092	0.813	453.86
0.05 mM	40.18	33.93	0.0012	0.799	537.89
0.005 mM	40.35	66.14	0.00099	0.813	529.41
0.002 mM	39.39	86.86	0.00098	0.805	541.79
0.0005 mM	40.40	322.80	0.00093	0.807	699.39
Pd NPs	39.75	286.05	0.00119	0.820	946.72

Table S5 Electrical parameters of the C_3N_4 and Pd SAs/C_3N_4 samples extracted from PEIS plots by fitting with an equivalent circuit model

Samples	R_s (Ω cm^2)	R_{ct} (Ω cm^2)	Q_0 (S)	n	C_t (μF cm^{-2})
C_3N_4	41.32	114.73	0.00073	0.854	485.28
10 mM	40.23	32.855	0.00131	0.728	408.94
2mM	40.78	38.694	0.00135	0.839	769.75
0.5 mM	40.86	38.463	0.00125	0.771	513.59
0.05 mM	40.42	52.203	0.00255	0.676	973.07
0.005 mM	40.73	52.600	0.00126	0.711	421.98
0.002 mM	42.24	59.703	0.00097	0.800	481.84
0.0005 mM	40.36	62.752	0.00092	0.771	395.77
Pd NPs	40.19	55.428	0.00116	0.734	435.37

References

1. A. Torres-Pinto, M. J. Sampaio, C. G. Silva, J. L. Faria and A. M. Silva, Metal-free carbon nitride photocatalysis with in situ hydrogen peroxide generation for the degradation of aromatic compounds, *Applied Catalysis B: Environmental*, 2019, **252**, 128-137.
2. C. Marchal, T. Cottineau, M. G. Méndez-Medrano, C. Colbeau-Justin, V. Caps and V. Keller, Au/TiO2–gC3N4 nanocomposites for enhanced photocatalytic H2 production from water under visible light irradiation with very low quantities of sacrificial agents, *Advanced Energy Materials*, 2018, **8**, 1702142.
3. M. Karimi-Nazarabad, H. Ahmadzadeh and E. K. Goharshadi, Porous perovskite-lanthanum cobaltite as an efficient cocatalyst in photoelectrocatalytic water oxidation by bismuth doped g-C3N4, *Solar energy*, 2021, **227**, 426-437.
4. S. Qin, J. Will, H. Kim, N. Denisov, S. Carl, E. Spiecker and P. Schmuki, Single atoms in photocatalysis: low loading is good enough!, *ACS Energy Letters*, 2023, **8**, 1209-1214.
5. Y. Wang, S. Qin, N. Denisov, H. Kim, Z. Bad'ura, B. B. Sarma and P. Schmuki, Reactive Deposition Versus Strong Electrostatic Adsorption (SEA): A Key to Highly Active Single Atom Co-Catalysts in Photocatalytic H2 Generation, *Advanced Materials*, 2023, **35**, 2211814.
6. S.-M. Wu, L. Wu, N. Denisov, Z. Badura, G. Zoppellaro, X.-Y. Yang and P. Schmuki, Pt Single Atoms on TiO2 Can Catalyze Water Oxidation in Photoelectrochemical Experiments, *Journal of the American Chemical Society*, 2024.
7. S. Mondal, L. Sahoo, M. Banoo, Y. Vaishnav, C. Prabhakaran Vinod and U. K. Gautam, Enhancing the Catalytic Activity of Pd Nanocrystals towards Suzuki Cross-Coupling by g-C3N4 Photosensitization, *ChemNanoMat*, 2024, **10**, e202300451.

8. N. Wang, J. Wang, J. Hu, X. Lu, J. Sun, F. Shi, Z.-H. Liu, Z. Lei and R. Jiang, Design of palladium-doped g-C3N4 for enhanced photocatalytic activity toward hydrogen evolution reaction, *ACS Applied Energy Materials*, 2018, **1**, 2866-2873.
9. Y. Akinaga, T. Kawawaki, H. Kameko, Y. Yamazaki, K. Yamazaki, Y. Nakayasu, K. Kato, Y. Tanaka, A. T. Hanindriyo and M. Takagi, Metal Single-Atom Cocatalyst on Carbon Nitride for the Photocatalytic Hydrogen Evolution Reaction: Effects of Metal Species, *Advanced Functional Materials*, 2023, **33**, 2303321.
10. L. Liu, X. Wu, L. Wang, X. Xu, L. Gan, Z. Si, J. Li, Q. Zhang, Y. Liu and Y. Zhao, Atomic palladium on graphitic carbon nitride as a hydrogen evolution catalyst under visible light irradiation, *Communications Chemistry*, 2019, **2**, 18.
11. R. Xu, B. Xu, X. You, D. Shao, G. Gao, F. Li, X.-L. Wang and Y.-F. Yao, Preparation of single-atom palladium catalysts with high photocatalytic hydrogen production performance by means of photochemical reactions conducted with frozen precursor solutions, *Journal of Materials Chemistry A*, 2023, **11**, 11202-11209.
